# The Relation between Neonatal Intensive Care Units and Postpartum Post-Traumatic Stress Disorder after Cesarean Section

**DOI:** 10.3390/healthcare11131877

**Published:** 2023-06-28

**Authors:** Eirini Orovou, Panagiotis Eskitzis, Irina Mrvoljak-Theodoropoulou, Maria Tzitiridou-Hatzopoulou, Maria Dagla, Christiana Arampatzi, Maria Iliadou, Evangelia Antoniou

**Affiliations:** 1 Department of Midwifery, University of West Attica, Agioy Spyridonos 28, 12243 Egaleo, Greece; 2 Department of Midwifery, University of Western Macedonia, Keptse, 50200 Ptolemaida, Greece; 3 Department of Psychology, National and Kapodistrian University of Athens, 15772 Athens, Greece

**Keywords:** neonatal intensive care unit, NICU, postpartum PTSD, birth trauma, cesarean section, hospitalized neonate, traumatic birth experience

## Abstract

Background: The experience of a neonate hospitalized in the NICU is an understandably traumatic experience for parents, especially for the mothers of neonates. This mental distress resulting from preterm birth and/or NICU hospitalization can be understood as post-traumatic symptomatology, according to the Diagnostic and Statistical Manual of Mental Disorders (DSM-5 version). The aim of this study is to investigate the impact of the admission of a neonate to the NICU (forany reason) on the development of postpartum PTSD in a sample of women after cesarean section.Methods: A total of 469 women who gave birth with cesarean section from July 2019 to June 2020 participated in this study out of the original sample of 490 women who consented to participate. Data were obtained from the researcher’s socio-demographic questionnaire and the post-traumatic stress checklist (PCL-5) from the Diagnostic and Statistical Manual of Mental Disorders DCM-5 version. Results: In total,11.7% of the sample experienced postpartum PTSD. There is a strong relationship between the inclusion of a neonate to the NICU due to perinatal stress, breathing difficulties, infections, and IUGR with postpartum PTSD (37.7%) in relation to the perinatal stress Criterion A (fear for the life of the neonate), the first criterion of postpartum PTSD. Conclusions: Additional measures must be taken for mothers of children who have been admitted to the neonatal intensive care unit with psychological support interventions and a reassessment of their mental state.

## 1. Introduction

The experience of a neonate hospitalized in aneonatal intensive care unit (NICU) is an understandably traumatic experience for the parents, while the feelings of mental distress may persist even after the neonate leaves the NICU [1]. Parents face separation from their child in an unfamiliar and stressful environment with access difficulties and when not receiving sufficient communication and information from the staff [2]. Such an experience can affect parental mental health, with an impact on the transition to parenthood. As one can perceive, for a woman to begin her parenting role inthe unfamiliar and stressful environment of the NICU is a truly traumatic experience, which is reinforced by possible breastfeeding difficulties. Early mother–infant separation increases the pressure and mental discomfort with respect tothe relationship between them. Mothers are mostly overwhelmed by feelings of shame, guilt, failure, and ambivalence, which are mainly due to social prejudices [3].

Of the studies that have so far assessed mental health outcomes in postpartum mothers, most describe mental health disorders that are strongly associated with the birth of preterm neonates [4]. Mental distress resulting from preterm birth and/or NICU hospitalization can be understood as part of post-traumatic symptomatology [5]. Post-traumatic stress (PTSD) symptoms include re-experiencing the event, increased reactivity, and avoidance and negative moods when at least one month has passed since the traumatic event [6,7]. It is already known that one in three women experience birth trauma [8], while about one in four can develop postpartum PTSD (P-PTSD) symptomatology [9]. Phillips [10] underlines the importance of the “magic hour”, i.e., the first minutes immediately after birth with respect to the breastfeeding mother–infant bond. Maintaining this contact after birth facilitates this bond and prevents the development of postpartum PTSD. Nevertheless, P-PTSD can affect the mother–infant bond [11,12,13] and increase the infant’s risks of developing behavioral or emotional problems later [14]. 

Several studies have demonstrated that many stressful events during the perinatal period, such as the pathology of gestation [15], emergency cesarean delivery [16,17], preterm birth [18], and postpartum complications [17], contribute to the development of P-PTSD [19]. In particular, emergency cesarean section (CS) has been linked to increased rates of maternal depression and PTSD in the postpartum period [20,21,22] due to unexpected events and the mother’s lack of mental preparation [23].

To meet the diagnosis of PTSD, exposure to a potentially stressful factor (Criterion A) must apply. More specifically, there must be exposure to actual or threatened death, serious injury, or sexual violence in any of the following ways: (a) direct exposure, (b) as a witness to the event, (c) learning that the traumatic event happened to a significant other, and (d) daily exposure through work [24]. Based on the above, admission to the NICU is considered a traumatic event for mothers who, in addition to a potentially traumatic birth experience such as a cesarean section, face the threat of death or physical unseemliness of the neonate. Mothers with PTSD have shown fearful behavior when interacting with their infants. These behaviors are more likely to be related to the avoidance symptom, where mothers avoid reminders of the traumatic event (inclusion of the neonate in NICU), which in this case could be the avoidance of the infant itself and its care. PTSD symptoms can also affect breastfeeding. Actually, re-experiences and avoidance symptoms are associated with the failure to initiate and maintain breastfeeding, while avoidance symptoms are associated with the inability to continue breastfeeding at 1 year postpartum [4]. In particular, according to Türkmen, there is a high correlation between P-PTSD, the traumatic perception of birth, and low breastfeeding self-efficacy. However, the most important reason for low breastfeeding rates in PTSD postpartum women is increased cortisol secretion, which suppresses oxytocin production, resulting in reduced milk production and the separation from the neonate, which is an important factor [25].

On the other hand, the prevalence of PTSD in the mothers of infants admitted to the NICU ranges around 40% according to previous studies [26,27]. Although there is great complexity among NICUs, as they consist of many departments comprising different levels of care (e.g., very seriously ill neonates or very premature, intermediate, and mild neonatal illness), parental stress appears to be high with respect to different departments and countries [28]. Parental stress appears to be independently associated with infant risk due to their NICU status because they are not always able to understand the magnitude and severity of neonatal illness. Moreover, mother–infant separation and its combination with the mother’s fear of its viability underlie her psychological distress in the postpartum period [29]. A recent study highlighted the importance of this separation, pointing to the sight of intravenous feeding tubes of medical machines and the inability to hold their child as strong factors in the development of mental injury [30]. Therefore, it is given that the mothers of hospitalized NICU neonates show greater levels of psychological discomfort than mothers of neonates that are non-admitted to the NICU [31].

So far, several studies have been conducted, and they mainly parental post-traumatic stress symptoms after the admission of a neonate to the NICU. However, NICU admission has not been studied as an additional trauma variable in the special population of postpartum women, such as women after CS. Furthermore, it is not known to what extent NICU may contribute to the high rates of PTSD in women after CS. Given that women who undergo CS (emergency or elective) are more likely to develop post-traumatic symptoms [16,17,20,32], the admission of the neonate to the NICU of CS-exposed women makes them a particularly vulnerable group of postpartum mothers. So far, there are several studies that show a correlation between cesarean section and PTSD [20,32]. More specifically, emergency cesarean sections play an important role in the development of postpartum mental disorders. Indeed, in this population of postpartum women, there are not only a greater number of depressive and post-traumatic symptoms but also a negative impact on the women’s perceptions of their childbirth [20]. Also, a positive relationship has been found between birth with CS and the admission of the neonate to the NICU [4,5]. However, there is no study that identifies the effect of the NICU on the special group of women after CS. 

Therefore, the purpose of this study is to investigate the impact of the admission of a neonate to the NICU (forany reason) on the development of P-PTSD in a sample of women after CS (planned or emergency).

## 2. Materials and Methods

The specific study was carried out between July 2019 and June 2020 at the obstetrics clinic of the University Hospital of Thessaly, Greece, with a prospective design. Our initial sample consisted of postpartum women after CS (planned and emergency) who were aged 18 or older and gave written informed consent to participate in the study. Our first sample consisted of 490 women who gave written consent for participation in the research study and met the predetermined criteria for participation in the study. From this sample, 21 women were lost after first contact, andfinally, 469 postpartum women responded to the follow-up and constituted the sample of our study.

### 2.1. Research Participation Criteria

The study was approved by the University Hospital of Larisa in Greece, Ethics Commission (Approval: 18838/08-05-2019).The research participants who gave birth with CS had a complete medical record at the specific hospital, which means that they were monitored during the entire pregnancy or most of it at the hospital, had a sufficient understanding of the Greek language, exhibited satisfactory mental levels, and were not taking psychotropic drugs or other substances during the perinatal period. The postpartum women who did not meet the above criteria were excluded from the study.

### 2.2. Measures and Data

Data were collected in 2 phases (2nd day and 6th week postpartum). During the first phase, questionnaires were given by the researcher to postpartum women on the second day after cesarean delivery during their hospitalization (socio-demographic; Criterion A) and after their informed consent, while data on each woman’s medical history were obtained. The place of approach and communication with the mothers was their hospital room during hours when there were no visitors and when no medical and nursing operations were performed; in the presence of other women, the mother was transferred to a quieter place. The second postoperative day coincides with women’s physical recovery from surgery, so it was chosen as the most appropriate day to conduct the first phase of the research.

The second phase took place during the sixth week postpartum. By conductinga telephone interview, the postpartum women answered questions about the existence of post-traumatic symptoms according to the PTSD scale (PCL-5). In order to be diagnosed with PTSD, it is necessary that a month has passed since the traumatic event, so this specific period (6th week) was considered appropriate to meet the diagnostic criteria [24]. All measures were received by the National Center of PTSD [6] and were translated into the Greek language and weighted by 2 research members (E.O. and E.A). For the needs of this research study, the van de Vijver and Leung [33] method was used, in which 2 bilingual independent obstetric care professionals translated the questionnaires from their original version (English language) into Greek. Finally, the pilot questionnaire was administered to a sample of 23 postpartum women before starting the main survey. The aim of the pilot administration of the questionnaires was to check the degree of understanding of the questions by all women regardless of age, socio-economic status, and educational level. Finally, PCL-5 showed very good psychometric properties during its evaluation. More specifically, the Cronbach’s α of this scale in our sample is 0.97 [34], which means that it can be used with respect to thesamples of postpartum women with very reliable results. 

#### 2.2.1. A researcher Socio-Demographic Questionnaire

This questionnaire included sections on the woman’s social characteristics, demographic data, and medical history, which included obstetric, gynecological psychiatric, and family information concerning the present situation; the neonate; and finally, specialized questions about the experience of birth with CS. 

#### 2.2.2. Stressor Perinatal Criterion A

Perinatal Criterion A is based on PTSD Criterion A [35] according to DSM 5. Criterion A is the first criterion, the existence of which is considered necessary for the other criteria to appear: B: re-experiencing; Criterion C: avoidance; Criterion D: negative alterations in cognitions and mood;Criterion E: hyperarousal and reactivity [6]. For the purposes of this study, Criterion A examined the population exposure of women following EMCS and ELCS. Therefore, Criterion A was adapted with appropriate specialized questions that diagnosed exposure to a maternal or fetal/neonatal life-threatening traumatic event. However, the Criterion A perinatal stressor was divided into the following: (a) Criterion A1, which detected maternal or child-threatening situations during or shortly before CS; and (b) Criterion A2, which detected complications in mother–child life after CS. 

#### 2.2.3. Post-traumatic Stress Checklist (PCL-5) [36]

The Post-traumatic Stress Checklist is a scale that assesses 20 post-traumatic symptoms according to DSM-5 and provides a provisional PTSD diagnosis. Postpartum women were asked to answer 20 self-report items that assessed 20 PTSD symptoms of Criteria: B (reexperiencing), C (avoidance), D (negative thoughts and feelings), and E (arousal and reactivity).

### 2.3. Statistical Analysis

Quantitative variables, describing the sample, are first presented mostly as frequencies and a mean value (*M&SD*) for the first variable in Table 1. Multivariate analysis of variance and linear regression analysis were performed to identify admission to the NICU that was associated with the presence of PTSD in postpartum women. Statistical significance was set at 0.05, and analyses were conducted using SPSS statistical software (SPSS Statistics version 22.0, IBM, Armonk, NY, USA).

## 3. Results

A total of 469 women were analyzed in this study. Their mean age was 32.58 ± 6.15 (*SD*) years, with the majority of them being from the city (*n* = 373, 79.5%); 98.5% (*n* = 462) were married, engaged, or in a relationship. Overall, 43.4% (*n* = 204) of the sample had completed undergraduate and/or postgraduate studies, with a similar percentage who had finished high school (*n* = 196, 41.8%); 10.7% (*n*= 69) participants had finished primary school or junior high. In relation to the reasons for admission to NICU, it can be observed that the most frequent reasons are prematurity (*n* = 49, 48.0%) and perinatal stress/breathing difficulty (*n* = 41, 40.1%) (Table 1). 

The next analysis conducted in the present study, with the independent variable admission to the NICU and the dependent PTSD, is a multivariate analysis of variance, together with the dependent variables being the four factors of PCL-5 (intrusion/re-experiencing, avoidance, negative alterations in cognitions and mood, and alterations in arousal and reactivity). Out of 469, 367 infants were not administrated to NICU. Prior to this, the correlations analysis among PCL-5 factors showed high and positive correlations ranging from 0.717 to 0.919. The results of the analyses of variance are presented in Table 2. The cut-off for the provisional PTSD diagnosis was 33.

At the univariate level, the *F* criterion showed a statistically significant relationship between admission to the NICU and all five independent variables. According to the Scheffépost hoc criterion, it is observed that mothers of the present sample, whose child was admitted to the NICU either because of prematurity or any other reason reported in Table 2, are more likely to develop PTSD (*p* < 0.001) and to have higher scores on intrusion/re-experiencing (*p* < 0.001), avoidance (*p* < 0.001), negative alterations in cognitions and mood (*p* < 0.001), and alterations in arousal and reactivity subscale (*p* < 0.001).

Finally, a linear regression analysiswas performed in order to investigate the relation between the reasons for the admission to the NICU, together with Criterion A, and PTSD as the dependent variable. Therefore, binary basis variables, also called dummy variables, are created to represent the independent variables; categorical independent variables cannot be directly entered into a regression analysis. Results are presented in Table 3.

Vialinear regression with PTSD being the dependent variable relating to the reasons for the admission to the NICU, which comprises Criterion A, apart from prematurity, the introduced model here and the other two predictors together are strongly related to PTSD: Criterion A (*β* = 0.51; *p* < 0.001) and perinatal stress/breathing difficulty/infection/IUGR (*β* = 0.11; *p* = 0.008). The percentage of variability interpreted by this model is statistically different from 0, with the regression equation reaching significance (*F* = 91.93, *df* = 3, *p* < 0.001, *R* = 0.584, and *R*^2^ = 0.337), which suggests that 33.7% of the variance of PTSD could be explained by this regression model. Consequently, the participants that experienced the situations appearing as a predictor in the present model were more likely to develop PTSD.

## 4. Discussion

The aim of this study was to investigate the impact of the admission of a neonate to the NICU (forany reason) on the development of P-PTSD in a sample of women after CS. Postpartum PTSD in the general postpartum population ranged between 3 and 27% [37,38,39], while women who undergo CS show percentages greater than 30% [17]. According to our results, 11.7%% of postpartum women after CS experienced P-PTSD. A percentage of 51% of women had experienced their CS as traumatic, while 52.2% of the total sample had no birth satisfaction (they did not expect to give birth in this way). However, 37.7% of postpartum women whose neonates were hospitalized due to perinatal stress, breathing difficulties, infections, and IUGR appeared to experience P-PTSD, while 38.8% comprised women whose neonates were admitted to the NICU due to prematurity. The high rate of PTSD is consistent with an earlier study in which PTSD was present in 60% of mothers whose children were admitted to the NICU [40]. Also, the results of another study [41] showed that the acute PTSD symptoms of postpartum mothers were related to having a neonate in the NICU forany reason.These results show high psychological trauma similar to those of major natural disasters [42,43,44], sexual assault [45], and war [46]. According to our results, mothers who underwent CS with children who were admitted to the NICU in combination with Criterion A (fear for the life of the neonate) due to perinatal stress, breathing difficulties, infections, and IUGR were more likely to develop P-PTSD than mothers whose children were admitted to the NICU due to prematurity. From what we have observed, prematurity was not a reason for the development of PTSD; therefore, this rate is due to other factors that could potentially threaten the life of the mother, such as emergency CS, complications, or dissatisfaction with the type of delivery.

To explain this phenomenon, we must emphasize that during an acute life-threatening event, the autonomic nervous system works to effectively respond to the threat [47]. However, acute psychological trauma that can lead to PTSD is associated with sudden events [6]. It seems, therefore, that prematurity may be related to other conditions that disrupt the smooth course of pregnancy and prepare the woman for a possible complication during childbirth [48,49].

On the other hand, all other reasons for admission to the NICU (infection, breathing difficulties, and perinatal stress) cannot always be foreseen, with the result that the mental preparation of the mother does not exist; therefore, the possibilityof mental injury increases. A recent published study [50] on PTSD among NICU mothers, showed that a majority of them (55%) and not only those with critically ill infants met the criteria for PTSD, a fact that shows that mental trauma is not related to the gravity of the event. 

Contrary to our results, there are many studies that focused only on critically ill or preterm neonates exhibiting low birth weight, prematurity, and infections [18,51,52,53] as causative factors in the development of postpartum PTSD. Additionally, the study of Lefkowitz [54] defends that several psychosocial factors contribute to the severity of post-traumatic symptoms. However, according to a recently published article [5], the gestational age of neonates admitted to the NICU significantly affects the occurrence of P-PTSD, while the lack of family support after the inclusion of the neonate inthe NICU was a high-risk factor for P-PTSD [55]. 

However, the P-PTSD of mothers who have children in the NICU is multifactorial. One of the important factors that may play a decisive role in the emergence of post-traumatic symptomatology is the parent’s perception of the severity of the illness. Actually, the degree of the severity of infantile illness does not always correspond to the perception of the severity of the illness. According to the Malin study [56], parents were more likely to perceive their infants as sick than the doctors and nurses who cared for them. Furthermore, within the NICU environment, the maternal role is diminished as there are strong restrictions governing the nursing and operating regulations of the unit. Additionally, the incubator and NICU environment may amplify the fears of mothers who already had a dysfunctional and maladaptive stress response [57]. As a result, prolonged mental distress in mothers often disruptsthe mother–infant relationship and also appears to affect children’s psychosocial development in the future [58,59].

## 5. Conclusions

According to our findings, the admission of a neonate to the NICU is an important factor in the development of P-PTSD in women after CS. However, prematurity in itself is not a risk factor since mothers are more psychologically prepared to respond to this event. The high incidence of PTSD in these women may be due to other factors. Since they did not meet perinatal stress Criterion A, they may be reliving an earlier mental trauma.

We saw that the impact of trauma can be subtle, insidious, or downright devastating. How an event affects an individual depends on many factors, including the characteristics of the individual, the type and characteristics of the event, developmental processes, the significance of the trauma, and psychosocial factors. Additional measures must be taken for mothers after CS and those whose children are admitted to the NICU with psychological support interventions and a reassessment of their mental state. Furthermore, special care is required to strengthen breastfeeding in order to reduce the intensity of the birth trauma and develop the mother–child bond. Finally, for further research, it would be interesting to explore which of the controlled socio-demographic variables may play the role of protective factors.

## Figures and Tables

**Table 1:** Demographic, perinatal, and mental health variables of the sample.

***Demographic Variables***	*f/M*	*rf/SD*
Age	32.58	6.15
Residence		
City	373	79.5
Village	96	20.5
Total	469	100.0
Nationality		
Greek	423	90.2
Other	46	9.8
Total	469	100.0
Family Status		
Single/Divorced	7	1.5
In a Relationship/Engaged	47	10.0
Married	415	88.5
Total	469	100.0
Educational Level		
Primary School	39	8.3
Junior High	30	6.4
High School	196	41.8
University	170	36.2
MSc/PhD	34	7.2
Total	469	100.0
Occupation		
Public/Private Sector	136	29.0
Freelance	67	14.3
Health Care Professional	38	8.1
Educators	43	9.2
Household	123	26.2
Unemployed	62	13.2
Total	469	100.0
Financial Status		
Low	136	29.0
Middle	320	68.2
High	13	2.8
Total	469	100.0
*Perinatal Health Variables*		
Parity		
0	202	43.1
1 birth	177	37.7
≥2 births	90	19.2
Total	469	100.0
Type οf Previous Labor		
No Previous Labor	204	43.5
Vaginal	41	8.7
C-section	224	47.8
Total/Missing	469	100.0
Kind οf Conception		
Normal	429	91.5
In Vitro Fertilization (IVF)	40	8.5
Total	469	100.0
Gynecologic History		
No	421	89.8
Yes	48	10.2
Total	469	100.0
Gestational Week	37.76	2.10
< 37	70	14.9
≥ 37	399	85.1
Total	469	100.0
Type οf C-section		
Emergency	181	38.6
Elective	288	61.4
Total	469	100.0
Cause οf C-section		
Previous c-section/Premature Rupture of Membranes/Premature Contractions in a Previous C-section/Placenta Previa	203	43.3
Abnormal Fetal Position	66	14.1
Twins/IVF gestation	30	6.4
Mother’s Desire	30	6.4
Heavy Medical History/Myopia/Previous Gynecological History/Preeclampsia	35	7.5
Failure of Labor to Progress	43	9.2
Abnormal Heart Rate/Pathological NST/Doppler/Premature Rupture of Membranes/Premature Contractions/Infection	62	13.2
Total	469	100.0
C-section Complications		
No	434	92.5
Yes	35	7.5
Total	469	100.0
Birth Expectations/Satisfaction		
No	245	52.2
Yes	224	47.8
Total	469	100.0
Reasons for Admission to the NICU		
Perinatal Stress/Breathing Difficulty	41	40.2
Infection	4	3.9
IUGR	2	2.0
Other Disorders	6	5.9
Prematurity	49	48.0
Total	102	100.0
Breastfeeding		
No	153	32.6
Yes	316	67.4
Total	469	100.0
*Mental Health Variables*		
Psychiatric History		
No	411	87.6
Yes	58	12.4
Total	469	100.0
Partner Support		
No	68	14.5
Yes	401	85.5
Total	469	100.0
Traumatic C-section		
No	230	49.0
Yes	239	51.0
Total	469	100.0
Criterion A1—Was your life or your child’s life in danger?		
No	368	78.5
Yes, of My Child	63	13.4
Yes, Mine	17	3.6
Yes, of Both of Us	21	4.5
Total	469	100.0
Criterion A2—Any complications involving you or your child?		
No	400	85.3
Yes, My Child	43	9.2
Yes, Me	16	3.4
Yes, Both of Us	10	2.1
Total	469	100.0
Criterion A		
No	345	73.6
Yes	124	26.4
Total	469	100.0
Diagnosis		
No Diagnosis	378	80.6
Partial PTSD	36	7.7
PTSD	55	11.7
Total	469	100.0
No admission to the NICU		
No Diagnosis	318	86.6
Partial PTSD	33	9.0
PTSD	16	4.4
Total	367	100.0
Perinatal Stress/Breathing Difficulty/Infection/IUGR		
No Diagnosis	31	58.5
Partial PTSD	2	3.8
PTSD	20	37.7
Total	53	100.0
Prematurity		
No Diagnosis	29	59.2
Partial PTSD	1	2.0
PTSD	19	38.8
Total	49	100.0

Note. *M* = Mean value; *SD* = standard deviation; *f* = frequency; *rf* = %.

**Table 2:** Multivariateanalysis of variance of admission to NICU in relation to PTSD and to PTSD subscales.

	Admission to the NICU						
	***NoAdmission to the NICU***	*Perinatal Stress/Breathing Difficulty/Infection/IUGR*	*Prematurity*				
	*M*	*M*	*M*	*F*	*df*	*p*	*η^2^*
PTSD	6.18 a	20.64 b	21.24 b	46.64	2	*p* < 0.001	0.388
Intrusion/ Re-experiencing	1.68 a	5.75 b	7.18 b	56.31	2	*p* < 0.001	0.195
Avoidance	0.72 a	2.26 b	2.20 b	31.58	2	*p* < 0.001	0.119
Negative Alterations in Cognitions and Mood	2.34 a	7.04 b	6.71 b	32.28	2	*p* < 0.001	0.122
Alterations in Arousal and Reactivity	1.44 a	5.58 b	5.14 b	41.31	2	*p* < 0.001	0.151

Note. Means that share a common index (a, b) do not differ significantly from each other according to the Scheffépost hoc criterion. *M* = mean value, *F* = *F* criterion, *df* = degress of freedom, *p* = *p*-value, *η*^2^ = eta squared value.

**Table 3:** Results of the linear regression analysis of the reasons for the admission to the NICU and Criterion Ain relation to PTSD.

	*b*	*S.E.*	*β*	*t*	*p*
(Constant)	3.863	0.618		6.255	<0.001
Criterion A	17.100	1.474	0.513	11.603	<0.001
NICU—Perinatal Stress/Breathing Difficulty/Infection/IUGR	4.846	1.821	0.105	2.661	0.008
NICU—Prematurity	2.831	2.046	0.058	1.383	ns

Note. *p* < 0.001, *b* = beta value, *S.E.* = stardard error, *β* = beta coefficient, *t* = *t*-value, *p* = *p*-value.

## Data Availability

Not applicable.
